# Habitat Heterogeneity Drives Niche Partitioning and Morphological Divergence in Two Parapatric *Diploderma* Lizard Species

**DOI:** 10.1002/ece3.73982

**Published:** 2026-07-06

**Authors:** Yuhao Wen, Songwen Tan, Yihua Xiang, Wei Gao, Peng Guo, Bingjun Dong, Yayong Wu

**Affiliations:** ^1^ College of Life Sciences Shenyang Normal University Shenyang China; ^2^ Faculty of Agriculture, Forest and Food Engineering Yibin University Yibin China; ^3^ Yibin Key Laboratory of Zoological Diversity and Ecological Conservation Yibin University Yibin China; ^4^ College of Life Sciences Tibet University Tibet China; ^5^ State Key Laboratory of Genetic Evolution & Animal Models, and Yunnan Key Laboratory of Biodiversity and Ecological Conservation of Gaoligong Mountain, Kunming Institute of Zoology, Chinese Academy of Sciences Kunming China

**Keywords:** *Diploderma*, habitat heterogeneity, Hengduan Mountains, interspecific differences, species adaptation

## Abstract

Understanding how closely related taxa diverge and persist in spatial contact zones is central to explaining biodiversity dynamics. Habitat heterogeneity can promote lineage divergence through disruptive ecological pressures that favor differentiation in morphology, behavior, and ecological traits. This study focused on two closely related parapatric species, *Diploderma yangi* and *D. slowinskii*, which occupy contrasting microhabitats across the Nujiang River Basin in western China. The analysis integrated environmental, morphological, and dietary datasets to elucidate how niche differentiation stabilizes species boundaries in a landscape characterized by pronounced heterogeneity. Altitudinal and microhabitat features, including substrate composition and stone size, clearly delineated their distributions. The arboreal species, *D. slowinskii*, exhibited a larger overall body size, elongated limbs, and an extended tail relative to the saxicolous‐terrestrial taxon *D. yangi* , consistent with biomechanical demands of climbing rather than ground‐based movement. Despite both taxa functioning as arthropod generalists, trophic profiles diverged markedly: *D. yangi* primarily consumes smaller prey, whereas *D. slowinskii* targets larger arthropods. This divergence in trophic niche breadth is potentially driven by habitat‐specific prey structure, a hypothesis that warrants further validation through assessments of prey abundance across varying habitats. These results suggest that strong environmental gradients within a narrow contact zone generate persistent ecological divergence, with coordinated shifts in habitat use, morphology, and diet. Collectively, this study supports a mechanistic model of parapatric diversification and underscores the role of fine‐scale habitat complexity in sustaining high species richness across the Hengduan Mountains.

## Graphical abstract

This study focused on two closely related parapatric species, Diploderma yangi and D. slowinskii, which occupy contrasting microhabitats across the Nujiang River Basin in western China. The analysis integrated environmental, morphological, and dietary datasets to elucidate how niche partitioning stabilizes species boundaries in a landscape characterized by pronounced heterogeneity. The findings supports a mechanistic model of parapatric diversification and underscores the role of fine‐scale habitat complexity in sustaining high species richness across the Hengduan Mountains.

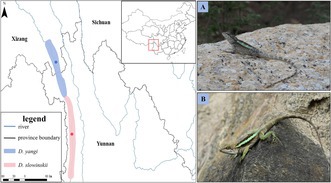

## 1 Introduction

Parapatric speciation refers to a mode of speciation in which two populations, distributed adjacent to but not overlapping in geographic range, gradually develop reproductive isolation through local adaptation and divergence. Unlike allopatric speciation, parapatric speciation allows for a certain level of gene flow; however, ecological selection pressure drives population differentiation along an environmental gradient (Butlin et al. 2008). Understanding the processes that govern divergence and stable coexistence among closely related taxa remains a central objective in evolutionary biology (Li et al. 2019). Such divergence often emerges from complex interactions among geographic structure, reproductive isolation, and ecological differentiation (Zhang et al. 2023), with habitat heterogeneity exerting a particularly profound influence by generating steep and spatially structured environmental gradients (Wiens 2024). These gradients reshape the ecological landscape by modifying resource availability, microhabitat configuration, and selective pressures, thereby driving differentiation in niche use and reducing interspecific competition (Begon et al. 2006). The persistence of species boundaries under such conditions depends on the coordinated coevolution of multiple adaptive traits, including morphological, behavioral, and dietary strategies (Xu et al. 2022; Bhatt and Lyngdoh 2023). Through complex interactions among topographic variation, vegetation architecture, and microclimatic factors, heterogeneous environments impose divergent filters that structure community composition and shape evolutionary trajectories (Moura et al. 2016; Janecke 2020). This environmental filtering, in turn, regulates species distributions (Laiolo 2013), promotes functional trait divergence (Deilmann et al. 2024), and drives shifts in trophic strategies (Cromsigt et al. 2009). These processes collectively reduce niche overlap and mitigate competitive exclusion, thereby facilitating the long‐term coexistence of closely related species across structurally complex landscapes (Bonsall et al. 2005; Mendes et al. 2017; Miller and Allesina 2023).

Habitat heterogeneity imposes divergent selective pressures on morphology by generating complex environmental gradients that vary across fine spatial scales (Marquart 2015; Khasanova et al. 2019). Interactions among topographic structure, substrate composition, and vegetation architecture produce pronounced microhabitat transitions that shape the evolutionary trajectories of functional traits under contrasting ecological constraints (Skeels 2020). For example, Johannesson's research on two distinct ecotypes of *Littorina saxatilis* (“crab morph” and “wave morph”) revealed significant morphological divergence between the two forms, which enables them to adapt to different environmental conditions (Johannesson 1986). This case provides clear evidence that habitat‐driven morphological differentiation facilitates niche segregation and limits interspecific competition, offering key insight into the mechanisms that support the coexistence of closely related species in structurally heterogeneous environments (Davis 2010; Zemouche 2018; Sanches et al. 2023).

Environmental heterogeneity also drives trophic divergence in foraging strategies by restructuring resource landscapes and modifying the spatial and temporal organization of interspecific interactions (Villsen et al. 2025). Shifts in prey availability and habitat structure can induce anatomical specialization of feeding apparatus (e.g., beak shape, dentition) and favor behavioral adaptations that reduce dietary overlap (Swanson et al. 2003, 2008). For example, In the Brazilian Coastal Plain, two parapatrically distributed subterranean rodent species, *Ctenomys minutus* and *C. flamarioni* , although both exhibiting a generalized diet, show preference differences for specific plant families (Lopes et al. 2015). Environmental DNA (eDNA) metabarcoding now enables high‐resolution detection of such cryptic dietary partitioning (DeSousa et al. 2019). However, investigations employing this technique to examine dietary niche differentiation among parapatric species remain relatively scarce, with most studies concentrating on sympatric or allopatric species distributions. Andriollo et al. (2021) used eDNA metabarcoding to compare the diets of three sympatric *Plecotus* bat species, which revealed significant interspecific differences in diet composition during spring but dietary convergence in summer and autumn, with habitat selection identified as the primary axis of dietary separation. Collectively, these studies underscore how environmental heterogeneity promotes multiaxis divergence that reduces competition and enables coexistence, reinforcing the role of spatiotemporal filtering in ecological speciation (Fujii et al. 2023).

Although interest in heterogeneity‐driven divergence has grown, most research has focused on taxa with short reproductive cycles, high phenotypic plasticity, or broad adaptive radiation, such as birds and fish (Dillon and Conway 2021). In contrast, lizards remain comparatively understudied, despite exhibiting remarkable functional diversity and habitat specialization. In particular, integrative analyses that link morphological and trophic differentiation in a shared environmental context are rare (Hagey et al. 2017). The parapatric sister species, *Diploderma yangi* and *D. slowinskii*, distributed across heterogeneous habitats in the Nujiang River Basin of western China, represent an ideal model system for investigating the ecological and evolutionary consequences of habitat filtering. Within their parapatric distribution ranges and sympatric range, *D. yangi* predominantly occupies shrublands, while *D. slowinskii* primarily inhabits open woodland. This study investigated how habitat heterogeneity drives synergistic differentiation in morphology and diet through integrated analysis of microhabitat structure, functional morphology, and fecal eDNA metabarcoding. The findings offer new insight into habitat‐driven divergence among closely related species in the Hengduan Mountains and clarify how environmental complexity shapes adaptive trait variation.

## 2 Materials and Methods

### 2.1 Survey Region

**FIGURE 1:**
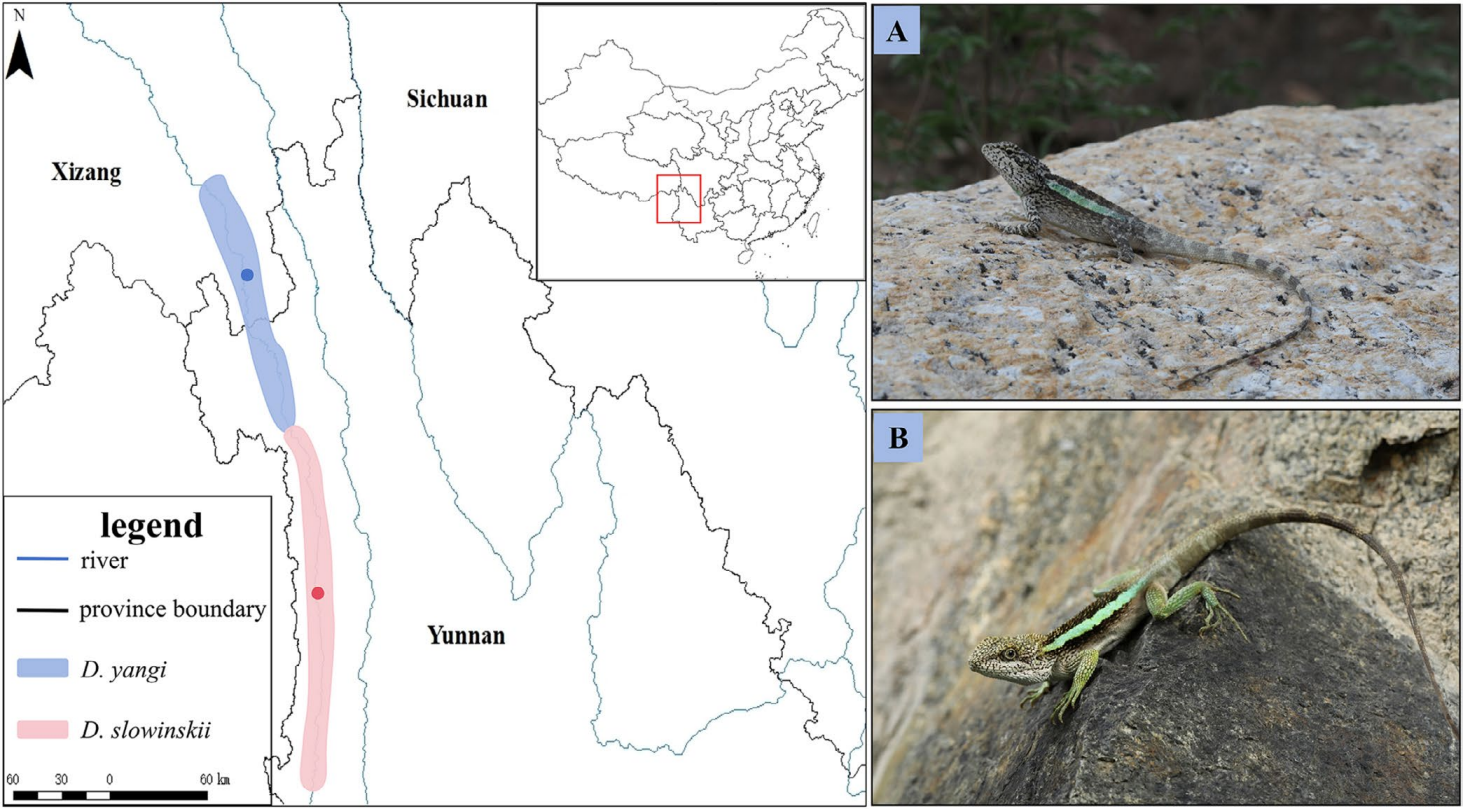
Distribution and habitat of *Diploderma yangi* and *D. slowinskii*. Left: Geographic ranges of *D. yangi* and *D. slowinskii*. Top‐right: *D. yangi* (A); bottom‐right: *D. slowinskii* (B). Distribution ranges are depicted as areas (patches), while sampling sites are indicated as points corresponding to the respective species. Species distribution range based on field survey data (unpublished data).

Fieldwork was carried out in the middle reaches of the Nujiang River in western China (Figure 1). *D. yangi* was surveyed in Cha'erlang Township, Zayu County, Tibet Autonomous Region (28.661° N, 97.467° E, elevation 1942 m), and *D*. *slowinskii* was surveyed in Shangpa Township, Fugong County, Yunnan (26.859° N, 98.869° E, elevation 1259 m). Both sites exhibit low levels of anthropogenic disturbance and high local population densities and are considered representative habitats for both species. Despite clear ecological differences, the two sites are separated by only 230 km.

### 2.2 Individual Capture and Measurement

A total of 120 adult *D. yangi* and *D. slowinskii* (30 males and 30 females per species) were sampled in August 2024. Individuals were captured by hand or using noose techniques during peak activity periods (10:00–12:30 and 15:00–18:00). Immediately after capture, habitat variables were recorded, and sex was determined based on dorsal coloration and the presence or absence of hemipenal bulges. Each lizard was assigned a unique identifier and temporarily housed in mesh cages for transport to a field laboratory. Morphometric measurements and fecal samples were collected, after which all individuals were released at their original capture location. Notably, all data were collected from adult specimens. Individuals of *D. yangi* with SVL > 50 mm and individuals of *D. slowinskii* with SVL > 70 mm were classified as adults.

### 2.3 Habitat Data Collection

For each lizard, a 5 m × 5 m plot was established centered at the point of capture. To assess available habitat, a control plot of equal dimensions was randomly positioned within the surrounding area using the random riprap method, maintaining a minimum distance of 10 m from the corresponding experimental plot. Any control plot containing a lizard was excluded and reassigned. Twelve ecological variables were quantified in each plot using appropriate field instruments, including eight continuous variables (temperature, humidity, altitude, perch height, vegetation coverage, vegetation height, stone height, and stone size) and four categorical variables (landscape habitat, habitat bottom material, discovery location, and vegetation type). Definitions and classifications followed Martín and Salvador (1997).

### 2.4 Morphological Data Collection

Morphological traits were recorded following standardized protocols (Slavenko et al. 2021). Nine traits were measured using a digital caliper (DL91200, Deli, Ningbo, China) with an accuracy of 0.01 mm, including snout‐vent length (SVL), tail length (TL), abdomen length (AL), head length (HL), head width (HW), head height (HH), snout length (SNL), forelimb length (FLL), and hindlimb length (HLL). Body mass (BM) was recorded using a digital scale (I‐2000, Mengfu, Dongguan, China) with a precision of 0.01 g.

### 2.5 Dietary Data Collection

Each lizard was housed individually in sterile, transparent disposable containers to allow for uncontaminated fecal collection. Fresh fecal material was collected immediately after defecation using sterile tweezers and preserved in 95% ethanol. Samples were labeled with species identity and a unique collection ID, then transported to the laboratory, where they were cryopreserved in sterile glycerol and stored at −20°C prior to DNA extraction.

Total genomic DNA was extracted from fecal material using a universal column‐based extraction kit. DNA concentration and integrity were assessed via 1% agarose gel electrophoresis. Two independent sets of PCR amplifications were conducted on dietary DNA extracts, employing primers targeting the chloroplast trnL (generate an amplicon of 120 bp) intron region and the mitochondrial COI (generate an amplicon of 100 bp) gene, respectively. For terrestrial plant detection, the primers trnL‐g (GGGCAATCCTGAGCCAA) and trnL‐h (CCATTGAGTCTCTGCACCTATC) were used; for terrestrial arthropods, the primers NoPlantF‐270 (RGCHTTYCCHCGWATAAAYAAYATAAG) and mlCOlintR‐W (GRGGRTAWACWGTTCAWCCWGTNCC) were used. To ensure consistent amplification across all samples, two criteria were maintained: (1) the number of amplification cycles was minimized, and (2) all reactions were run with an identical number of cycles.

All samples were processed under the formal experimental conditions, with three biological replicates per sample. The PCR products from the same sample were pooled and analyzed by 2% agarose gel electrophoresis. The PCR products were then recovered from the gel using the AxyPrep DNA Gel Extraction Kit (Axygen), eluted with Tris–HCl buffer, and reverified by 2% agarose gel electrophoresis. Polymerase chain reaction (PCR) amplification was performed using TransStart FastPfu DNA Polymerase in a 20 μL reaction system (4 μL of 5 × FastPfu Buffer, 2 μL of 2.5 mM dNTPs, 0.8 μL of 5 μM forward primer, 0.8 μL of 5 μM reverse primer, 0.4 μL of FastPfu polymerase, 10 ng of template DNA, and ddH_2_O to a final volume of 20 μL). Thermocycling conditions were as follows: initial denaturation at 95°C for 5 min, followed by 30 cycles of denaturation at 95°C for 30 s, annealing at 58°C for 30 s, and extension at 72°C for 45 s, concluding with a final extension at 72°C for 10 min. Based on preliminary electrophoresis quantification results, PCR products were quantified using the QuantiFluor‐ST Blue fluorescence system (Promega Corporation). Amplicons were pooled in equimolar concentrations based on sequencing requirements. Following this, library construction and sequencing were conducted on the Illumina MiSeq platform with paired‐end 300 bp (PE300) reads. Library construction protocol: (1) Ligate *Y*‐shaped adapters, (2) Remove adapter self‐ligation fragments using magnetic bead selection, (3) Enrich library templates using PCR amplification, (4) Denature with sodium hydroxide to generate single‐stranded DNA fragments; The sequencing methodology proceeds as follows: (1) One end of the DNA fragment is complementary to the primer bases and is immobilized on the chip, (2) The opposite end randomly hybridizes with a complementary primer from a neighboring site, which is also immobilized, thereby forming a “bridge” structure, (3) PCR amplification generates DNA clusters, (4) Denaturation of double‐stranded DNA amplicons into single‐stranded form, (5) Modified DNA polymerase and four types of fluorescently labeled reversible terminator dNTPs are added, with only a single base synthesized per cycle, (6) Laser scanning of the reaction plate surface to detect the type of nucleotide incorporated during the first round of polymerization for each template sequence, (7) Chemical cleavage of the fluorescent dye and terminator moiety to restore the reactive 3′‐hydroxyl group, enabling continuous polymerization of the subsequent nucleotide, (8) Statistically analyze the fluorescent signals collected in each cycle to determine the sequence of the template DNA fragments. PCR amplification and sequencing were performed by Shanghai Biozeron Biotechnology Co. Ltd. (China). The raw sequencing reads are processed through the following steps: (1) Filter bases at the 3′ end of reads with quality scores below Q20 using a 10 bp sliding window. If the average quality score within the window drops below Q20, trim from the window position to the 3′ end. Finally, remove reads shorter than 50 bp after quality control; (2) Merge paired‐end reads into a single sequence based on the overlap relationship between reads, with a minimum overlap length of 10 bp; (3) The maximum allowable mismatch rate for the overlap region of stitched sequences is 0.2; sequences that do not meet this criterion are filtered out; (4) Samples were demultiplexed based on the barcodes and primers flanking the target regions, with sequence orientation corrected accordingly. Barcode error tolerance was set to 0 mismatches, while primer mismatch tolerance was set to a maximum of 2 mismatches; (5) Remove chimeras using Usearch software and the GOLD database, combining de novo and reference‐based approaches. Finally, Divisive Amplicon Denoising Algorithm 2 (DADA2) is employed to partition all distinct sequences. A sequence with the highest abundance is first designated as the central sequence. All partitions are then compared against the central sequence, and the *p*‐values for abundance are calculated. The *p*‐value quantifies the probability that a sequence is determined to be an amplification error. If the minimum *p*‐value falls below the predefined threshold, a new partition is generated. All sequences are subsequently compared with the new partition, and *p*‐values are recalculated. This iterative process continues until each sequence is assigned to the most probable partition. Subsequently, Amplicon Sequence Variants (ASVs) are used to construct a Feature table, enabling further data filtering to remove low‐quality reads and chimeras, resulting in the generation of a feature table. To obtain species identification information for each amplicon sequence variant (ASV), we first filtered Arthropoda and Plants from the NT database to construct a local database, and then performed taxonomic analysis on the representative ASV sequences using either the uclust algorithm (confidence threshold set at 0.8) or the BLASTn alignment method (*E*‐value threshold set at 1e‐10).

### 2.6 Data Analysis

All variables were log‐transformed prior to analysis to improve normality, and missing data were imputed using multiple imputation by chained equations (MICE). Distributions were assessed for normality and homogeneity of variance, with Box‐Cox transformations applied when assumptions were violated.

To assess sex‐related variation in habitat use, principal component analysis (PCA) was first applied to evaluate habitat differences between males and females across both species. Based on the results, the sexes were merged for subsequent analyses. With the exception of altitude, all remaining habitat variables were included in subsequent analyses. To examine whether each species exhibits specific habitat selection preferences, differences between selected and control plots were analyzed using Wilcoxon test and chi‐square tests. And then, Wilcoxon test and chi‐square tests were performed on each habitat variable to test for interspecific differences between *D. yangi* and *D. slowinskii*. Highly correlated variables were excluded based on autocorrelation analysis. The remaining continuous habitat variables for both species were then combined to construct a random forest model, where *D. yangi* was assigned a value of 1 and *D. slowinskii* was assigned a value of 0.5. Partial dependence plots were generated to visualize species‐specific responses to habitat variables, with a partial dependence value of 0.5 (the median) serving as the threshold to separate *D. yangi* and *D. slowinskii*, and variable importance was quantified using the Mean Decrease in Gini index (Zhang et al. 2020). Additional analyses—including PCA, permutational multivariate analysis of variance (PERMANOVA; ADONIS), and analysis of similarities (ANOSIM)—were performed to assess overall habitat selection differences between *D. yangi* and *D. slowinskii*.

To examine morphological differences in *D. yangi and D. slowinskii* across different sexes, principal component analysis (PCA) was initially conducted to assess sexual dimorphism within the species, thereby facilitating subsequent interspecific comparisons between males and females separately. To examine differences in morphological traits between the two species, one‐way analysis of variance was employed. Additionally, PCA and permutation multivariate analysis of variance (PERMANOVA) were utilized to investigate significant morphological differences in the overall forms of *D. yangi* and *D. slowinskii*.

To compare dietary habits between the two species, we first integrated local insect diversity surveys and excluded taxa with relative abundance less than 0.1%. Subsequently, species composition and β‐diversity analyses were conducted on the ASV (Amplicon Sequence Variant) table obtained from filtered fecal DNA. *D. yangi* was designated as Group A and *D. slowinskii* as Group B for the assessment of trophic niche differentiation, facilitating subsequent interspecific comparisons. A chord diagram was employed to visualize the relative abundance of dietary categories and to compare differences in dietary composition and relative abundance between the two groups. To examine the dietary differences between *D. yangi and D. slowinskii*, nonmetric multidimensional scaling (NMDS) analysis was applied to conduct β‐diversity analysis between the two groups and evaluate differences in their dietary composition.

Analyses were performed in R v4.2.3 and SPSS v27. Data visualization was carried out using R v4.2.3 and Origin 2022. Unless otherwise indicated, all figures were produced using the aforementioned software. Analysis was conducted with a confidence level of 0.05.

## 3 Results

Morphological and habitat data were collected from 30 individuals of each species. Dietary data were obtained from 25 *D. yangi* individuals and 10 *D. slowinskii* individuals. The related eDNA data have been publicly deposited in the National Center for Biotechnology Information under the accession number PRJNA1399861. Morphological data and habitat data were both uploaded as attachments to the submission system.

### 3.1 Habitat Differences

**FIGURE 2:**
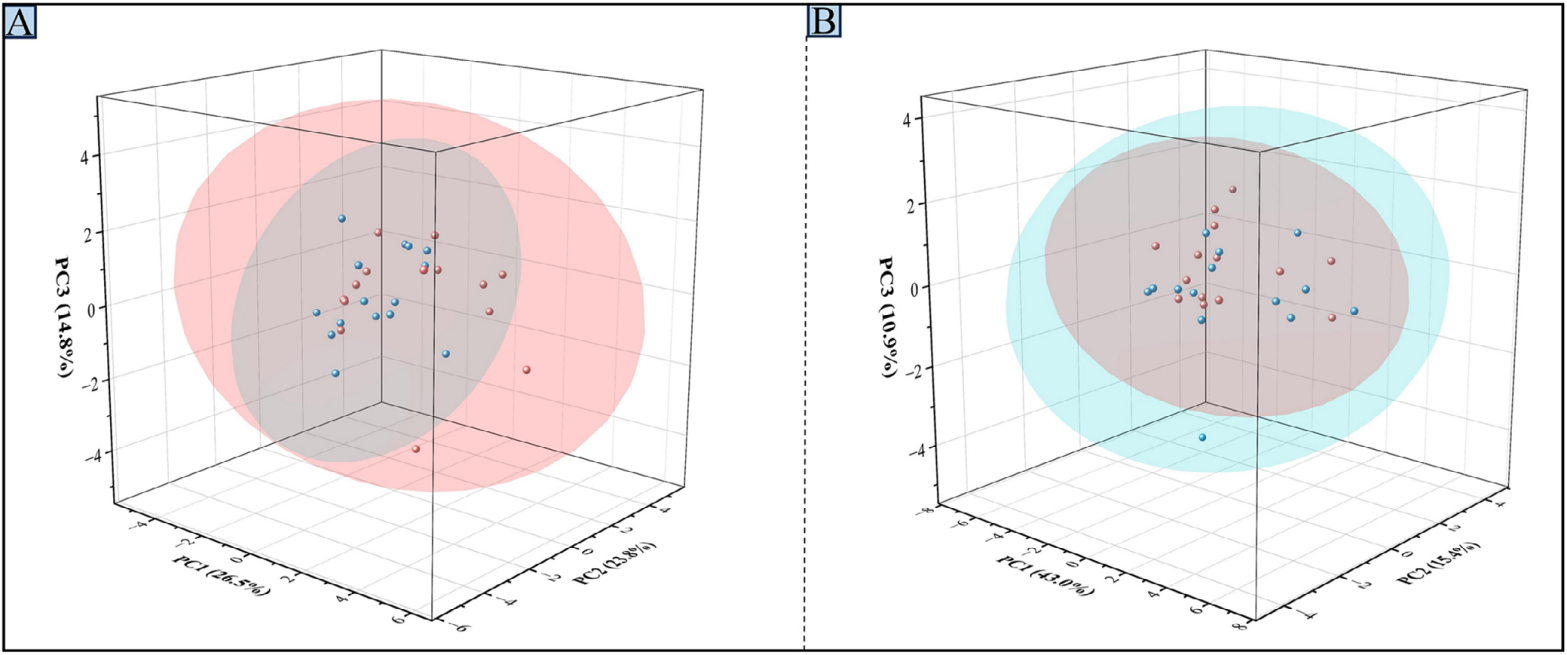
PCA Analysis of Intraspecific Habitat Differentiation between *Diploderma yangi* and *D. slowinskii*. (A) *D. yangi* ; (B) *D. slowinskii*. Blue: Females; Red: Males.

Prior to the interspecific analysis of *D. yangi* and *D. slowinskii*, a Principal Component Analysis (PCA) was performed on intraspecific males and females of both species. The analysis results indicated that no significant differences were observed among males and females of both *D. yangi* and *D. slowinskii* (Figure 2). Therefore, the male and female individuals of both species were pooled together for subsequent interspecific analysis.

The analysis of habitat preferences indicated that both *D. yangi* and *D. slowinskii* exhibited significant habitat preferences for certain habitat variables (Table S1). Further analyses revealed marked ecological separation between *D. yangi* and *D. slowinskii*. Notably, *D. yangi* primarily occupied arid, high‐altitude river valleys dominated by shrubland, while *D. slowinskii* mainly occurred in tall broadleaf forests.

**FIGURE 3:**
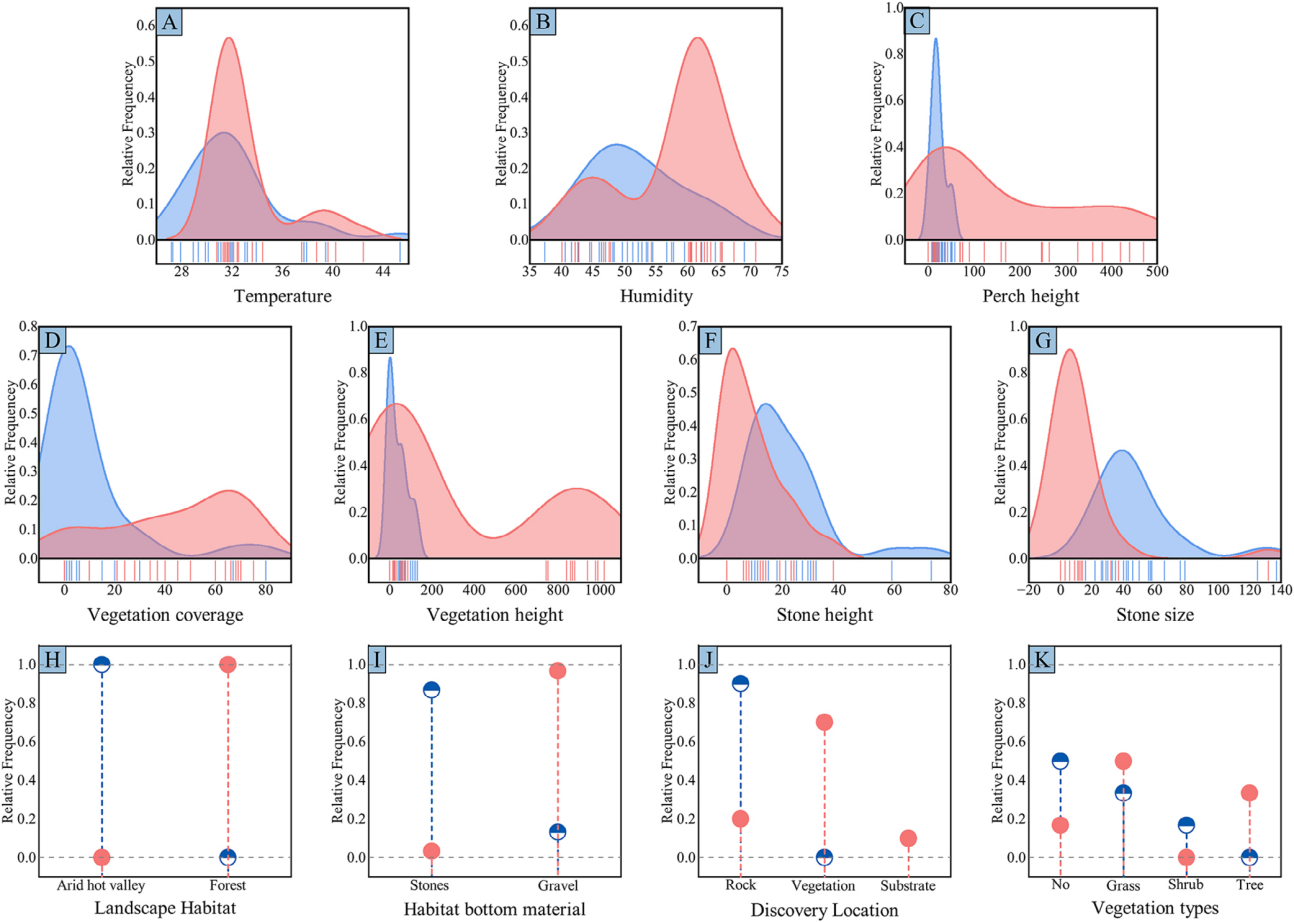
Habitat selection differences between *Diploderma yangi* and *D. slowinskii*. (A) Interspecies comparison of temperature; (B) Interspecies comparison of humidity; (C) Interspecies comparison of perch height; (D) Interspecies comparison of vegetation coverage; (E) Interspecies comparison of vegetation height; (F) Interspecies comparison of stone height; (G) Interspecies comparison of stone size; (H) Interspecies comparison of landscape habitat; (I) Interspecies comparison of habitat bottom material; (J) Interspecies comparison of discovery location; (K) Interspecies comparison of vegetation types. Blue: *D. yangi* ; Red: *D. slowinskii*.

Statistical comparisons identified significant interspecific differences in 10 of the 11 measured environmental variables, including humidity (*W* = 258.5, *p* = 0.005), perch height (*W* = 239.0, *p* = 0.002), vegetation coverage (*W* = 164.5, *p* < 0.001), vegetation height (*W* = 287.0, *p* = 0.014), stone height (*W* = 716.0, *p* < 0.001), stone size (*W* = 853.0, *p* < 0.001), landscape habitat (*p* < 0.001), habitat bottom material (*p* < 0.001), discovery location (*p* < 0.001), and vegetation types (*p* < 0.001). Temperature was the only factor that did not differ significantly between the species (*W* = 357.0, *p* = 0.17; Figure 3).

**FIGURE 4:**
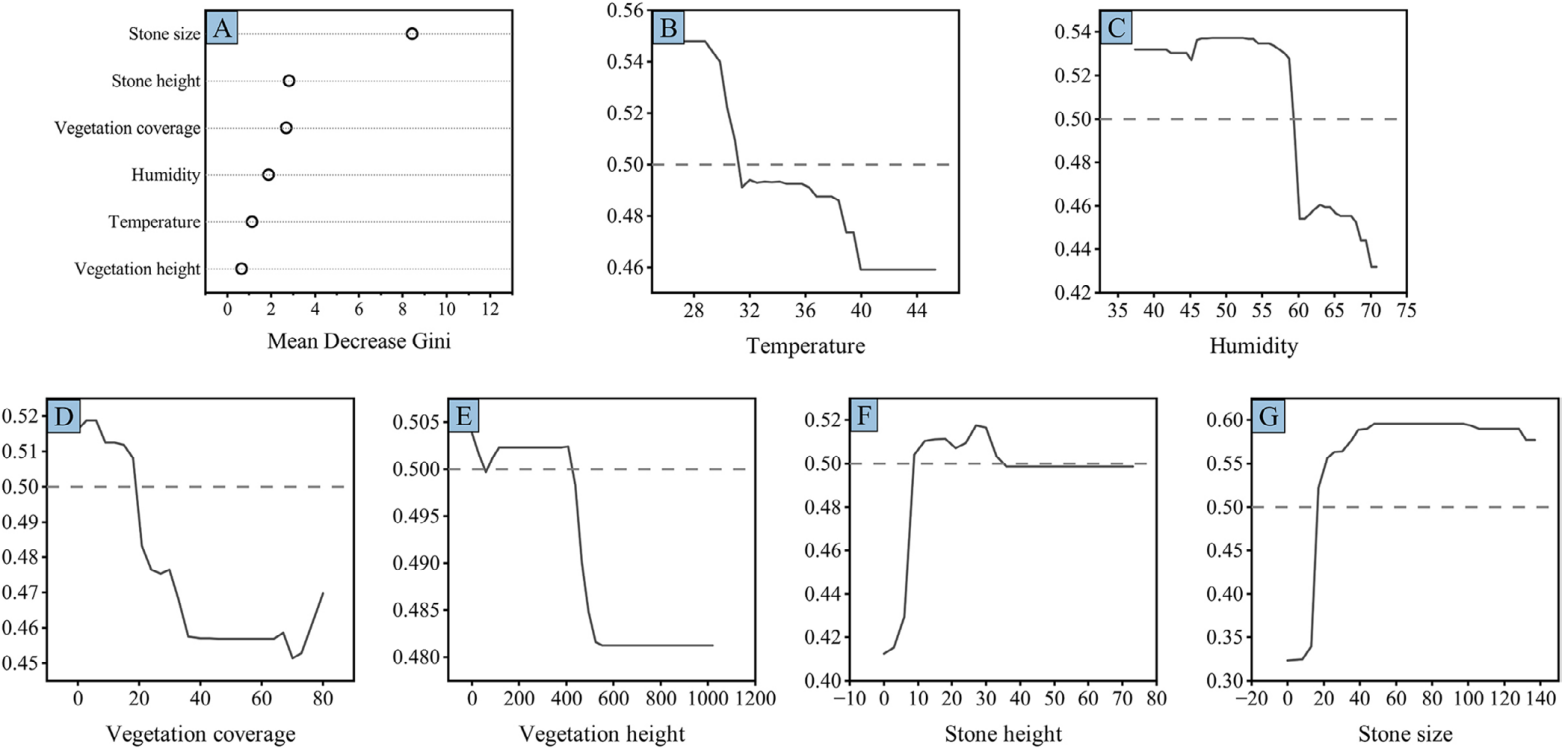
Variable importance and partial dependence plots for each predictor variable. (A) Importance scores for each predictor variable. (B–G) Partial dependence plots showing species occurrence probability as a function of each variable.

The random forest model trained on the combined habitat dataset identified altitude and stone size as the most discriminative variables, followed by stone height, vegetation coverage, and humidity. In contrast, temperature and vegetation height had minimal impact (Figure 4A). Partial dependence plots further visualized the differences in habitat preferences between the two species across each habitat variable. Specifically, regions with partial dependence values below 0.5 correspond to the preferred range of *D. slowinskii*, whereas peak regions above 0.5 indicate the preferred range of *D. yangi* (Figure 4B–G).

**FIGURE 5:**
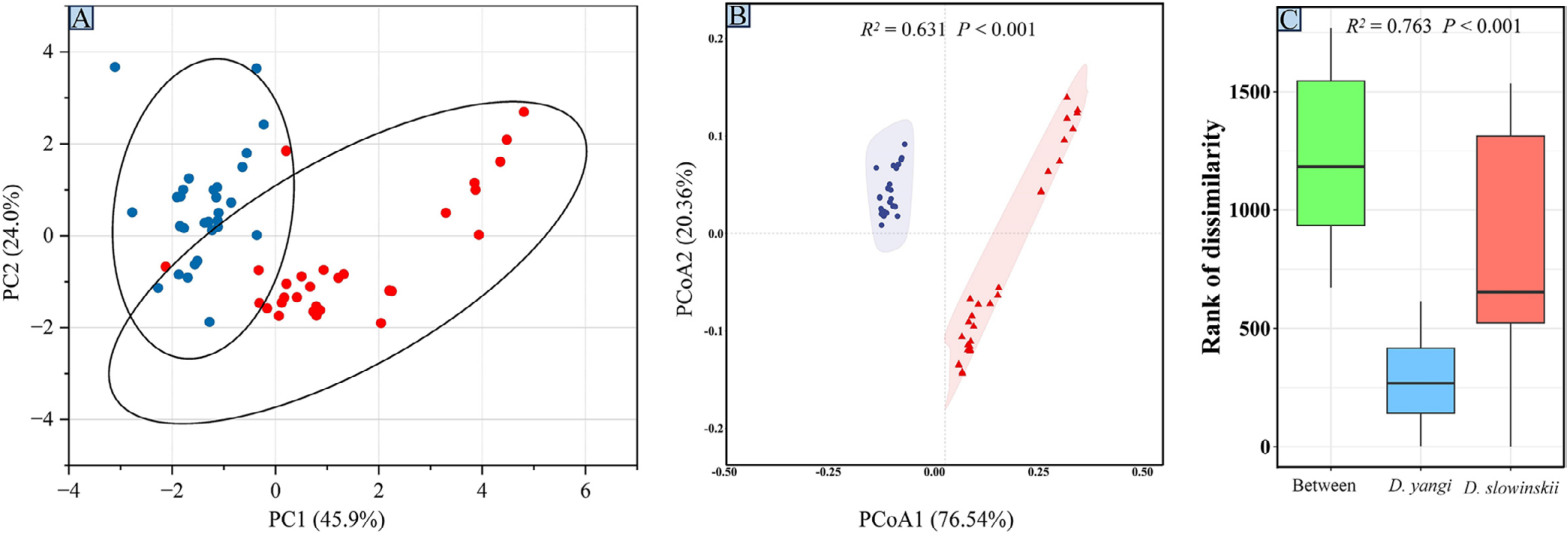
Overall differences in habitat selection between *Diploderma yangi* and *D. slowinskii* based on PCA (A), ADONIS (B), and ANOSIM (C). Blue: *D. yangi* ; Red: *D. slowinskii*; Green: Interspecific relationship.

PCA also revealed clear differences in habitat selection between *D. yangi* and *D. slowinskii* (Figure 5A), which was statistically supported by ADONIS (*R* ^2^ = 0.631, *p* < 0.001, Figure 5B). ANOSIM further demonstrated that the interspecific habitat differences between *D. yangi* and *D. slowinskii* were significantly greater than intraspecific differences (*R* ^2^ = 0.763, *p* < 0.001). Furthermore, compared to *D. slowinskii*, *D. yangi* exhibited more consistent intraspecific habitat preferences (Figure 5C).

### 3.2 Morphological Differences

**FIGURE 6:**
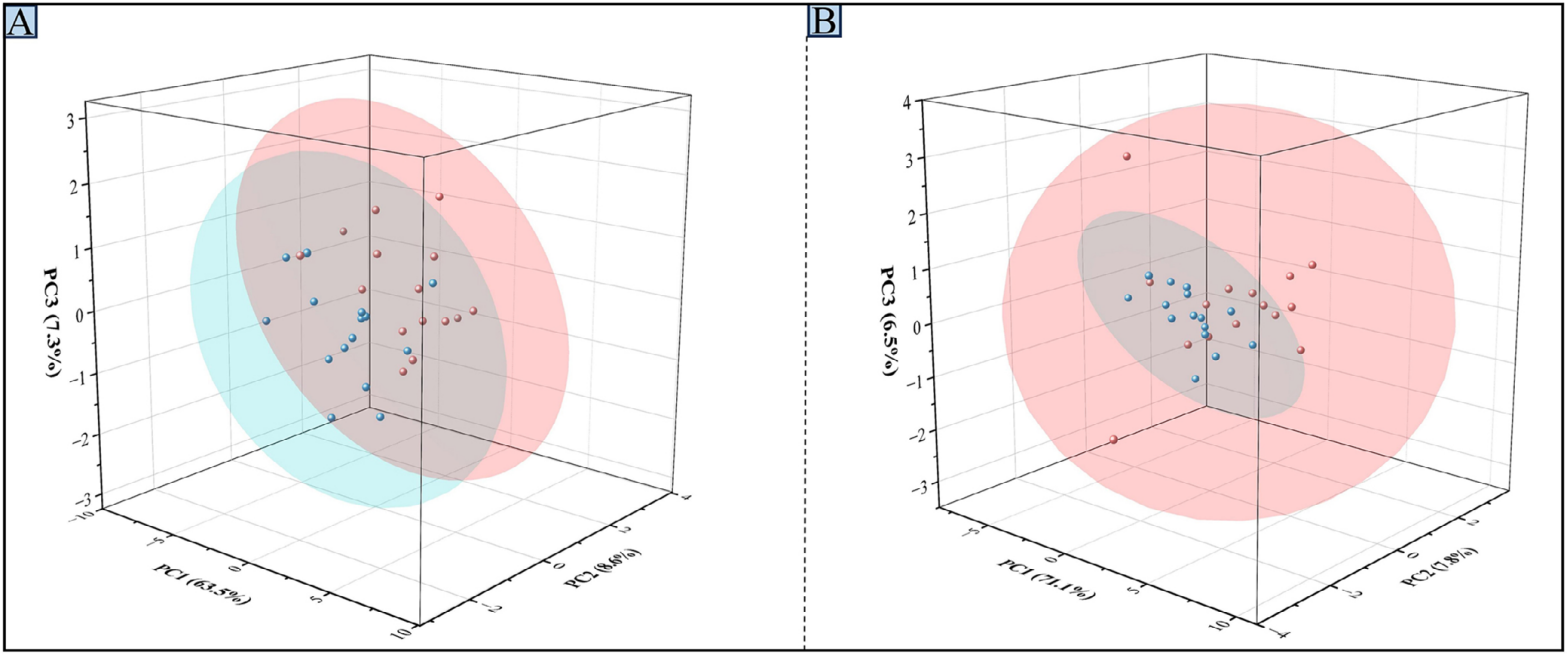
PCA Analysis of Intraspecific Morphological Differentiation between *Diploderma yangi* and *D. slowinskii*. (A) *D. yangi* ; (B) *D. slowinskii*. Blue: Females; Red: Males.

First, a Principal Component Analysis (PCA) was conducted for intraspecific comparison between sexes of *D. yangi* and *D. slowinskii*. The results showed that *D. yangi* exhibited obvious sexual dimorphism, whereas although *D. slowinskii* also presented sexual dimorphism, the morphological differences between male and female individuals were relatively minor (Figure 6, Table S2). Therefore, during interspecific analysis of the two lizard species, separate analyses were performed according to sex: male individuals of both dragon lizards were compared independently, and female individuals were compared separately.

**FIGURE 7:**
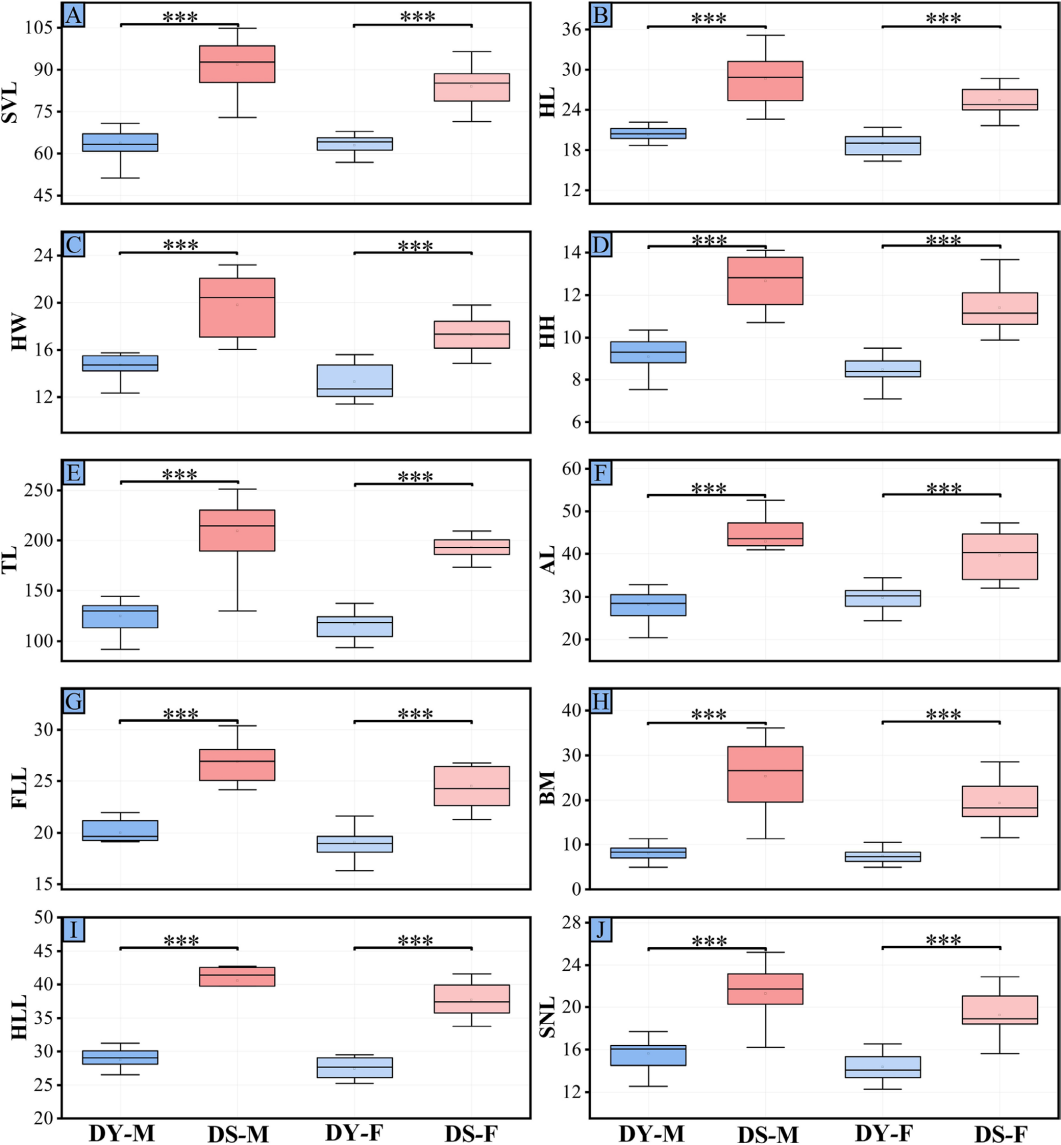
Interspecific Morphological Comparison between *Diploderma yangi* and *D. slowinskii*. (A) Comparative analysis of snout‐vent length (SVL); (B) Comparative analysis of tail length (TL); (C) Comparative analysis of abdomen length (AL); (D) Comparative analysis of head length (HL); (E) Comparative analysis of head width (HW); (F) Comparative analysis of head height (HH); (G) Comparative analysis of snout length (SNL); (H) Comparative analysis of forelimb length (FLL); (I) Comparative analysis of hindlimb length (HLL); (J) Comparative analysis of body mass (BM). DY‐M: ***D. yangi*** males; DS‐M: *D. slowinskii* males; DY‐F: ***D. yangi*** females; DS‐F: *D. slowinskii* females. *p < 0.05, **p < 0.01, ***p < 0.001.

**FIGURE 8:**
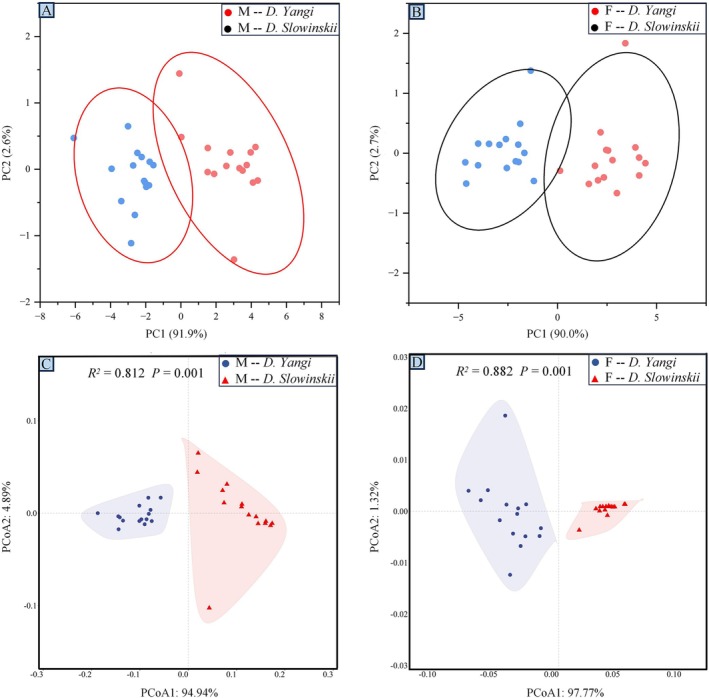
PCA and PERMANOVA analyses of morphological traits in *Diploderma yangi* and *D. slowinskii*. (A) Principal Component Analysis of interspecific males; (B) Principal Component Analysis of interspecific females; (C) Principal Coordinate Analysis of interspecific males; (D) Principal Coordinate Analysis (PCoA) of interspecific females.

Morphological comparisons based on 30 individuals per species demonstrated consistent interspecific divergence. One‐way ANOVA revealed that both male and female *D. slowinskii* exhibited significantly greater values across all measured traits relative to *D. yangi* , including HL (*F* = 100.40, *p* < 0.001; Figure 7, Supplementary Table 3), HH (*F* = 91.20, *p* < 0.001), HW (*F* = 59.50, *p* < 0.001), SNL (*F* = 46.28, *p* < 0.001), TL (*F* = 300.90, *p* < 0.001), AL (*F* = 71.10, *p* < 0.001), FLL (*F* = 73.67, *p* < 0.001), HLL (*F* = 195.10, *p* < 0.001), SVL (*F* = 121.10, *p* < 0.001), and BM (*F* = 140.20, *p* < 0.001). PCA and PERMANOVA further confirmed that significant morphological differentiation was observed between the two species, *D. yangi* and *D. slowinskii*, in the same‐sex individuals (*p* = 0.001, Figure 8).

### 3.3 Dietary Differences

Following dietary analysis using metabarcoding technology, to ensure the accuracy of dietary data and in conjunction with local insect diversity survey results, food units with a relative abundance below 0.1% were excluded, and the distribution patterns of the remaining food units were characterized. The results indicated that both *D. yangi* and *D. slowinskii* primarily consumed arthropods as the main food resource, with plants as a secondary food resource, classifying them as omnivorous species. However, significant differences were observed in their taxonomic composition.

**FIGURE 9:**
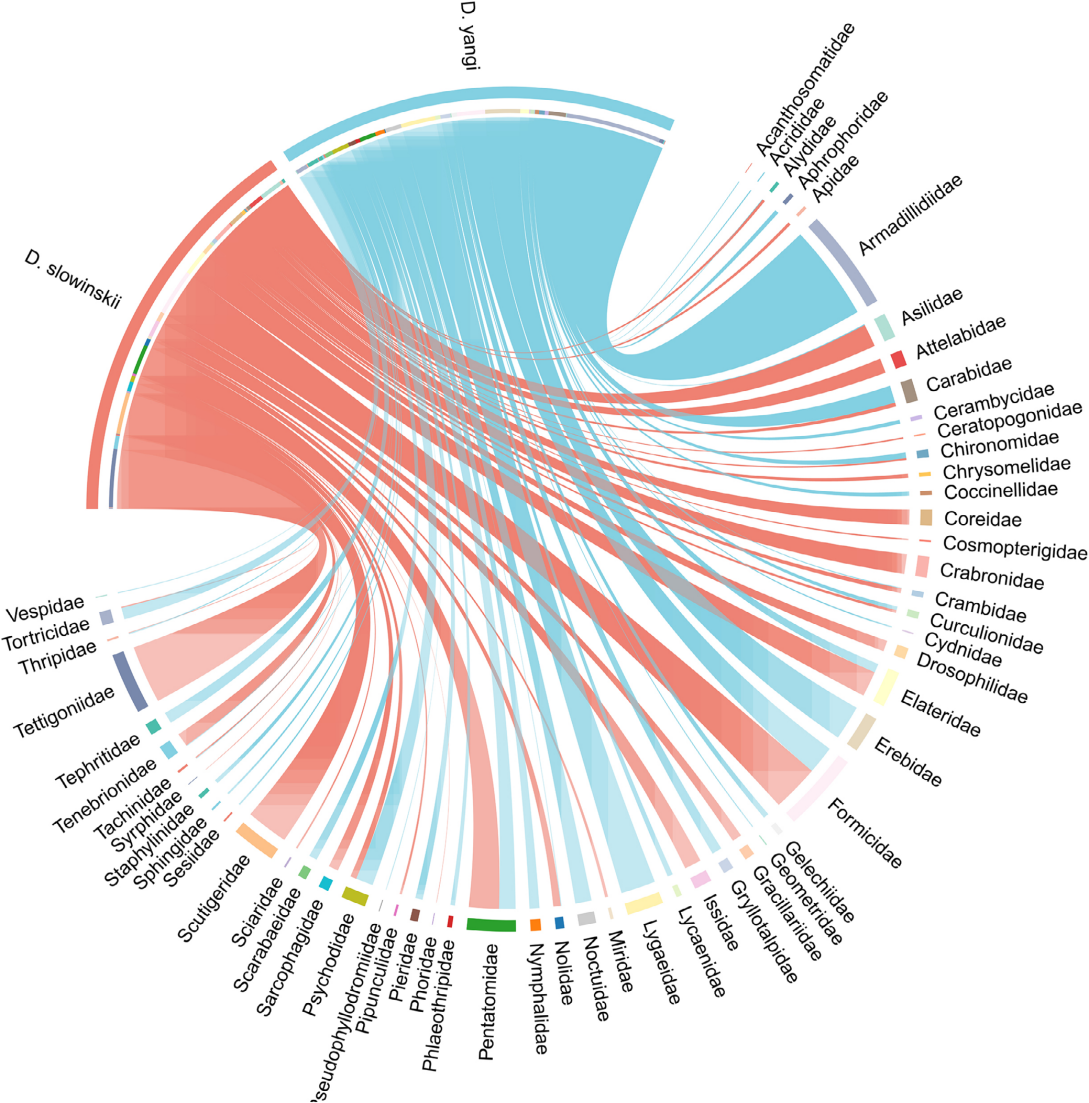
Chord Diagram Displaying Insect Prey Composition and Relative Abundance by Family for *Diploderma yangi* and *D. slowinskii*. Blue: ***D. yangi*** ; Red: *D. slowinskii*.

**FIGURE 10:**
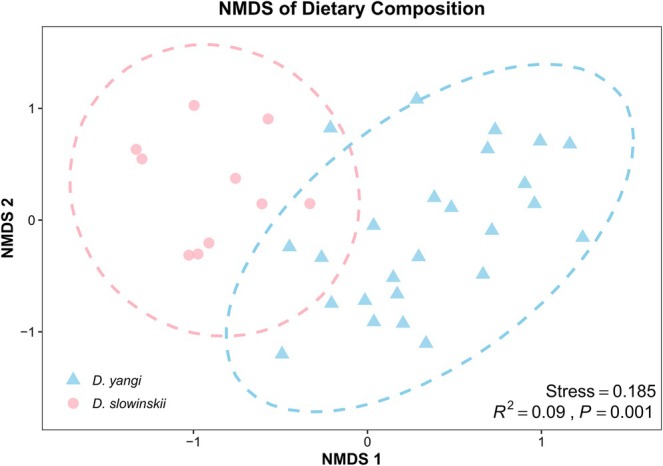
NMDS plot of dietary composition of *Diploderma yangi* and *D. slowinskii*. Blue: *D. yangi* ; Red: *D. slowinskii*.

Notably, *D. yangi* preyed on arthropods from 2 classes, 9 orders, and 39 families, with Armadillidiidae (24.69%), Lygaeidae (8.98%), and Erebidae (8.98%) as dominant taxa. In contrast, *D. slowinskii* consumed arthropods from 2 classes, 8 orders, and 33 families, primarily Tettigoniidae (15.01%), Formicidae (12.12%), and Scutigeridae (10.87%). Statistical analysis confirmed significant trophic niche differentiation. Notably, in terms of food items with relative abundance exceeding 5%, *D. yangi* primarily consumed small to medium‐sized insects (length < 50 mm), whereas D. slowinskii consumed both small and relatively larger arthropods (length ≥ 50 mm); moreover, from the overall dietary composition perspective, *D. yangi* exhibited a broader trophic niche (Figure 9, Table S4). Both NMDS demonstrated clear segregation in dietary composition between the two species (Stress = 0.185, *R* ^2^ = 0.09, *p* = 0.001; Figure 10), indicating distinct patterns of ecological niche exploitation.

## 4 Discussion

Environmental heterogeneity functions as a powerful ecological driver of adaptive divergence in morphology and trophic behavior, enabling organisms to exploit spatially variable resources and navigate distinct survival pressures (Bolnick et al. 2003; Fujii et al. 2023). Divergent selection across heterogeneous landscapes promotes phenotypic and behavioral differentiation among closely related taxa, facilitating ecological partitioning across microhabitats (Johnson and Wade 2010; Gabelaia et al. 2018). This conclusion was corroborated by studies on parapatrically distributed species, *Feirana quadranus* and *F. taihangnica* . The findings revealed that *F. quadranus* exhibits larger body size than *F. taihangnica* . In transitioning from allopatric to sympatric distribution, the two species diverged in pedal and manual morphological traits, while converging in eye diameter and interorbital distance. This research provides scientific evidence and theoretical support for understanding the potential evolutionary processes of neighborhood‐distributed species driven by interspecific competition and local adaptation (Huang et al. 2020). The present study integrated multidimensional data, including habitat preferences, morphological traits, and trophic niches, to systematically investigate patterns of interspecific divergence between two closely related *Diploderma* species in the Nujiang River Basin. Results revealed significant divergence in habitat utilization between the two species, with *D. yangi* primarily inhabiting low‐shrub environments in arid plateau valleys and *D. slowinskii* predominantly occupying broadleaf forests characterized by tall arboreal vegetation. Random forest modeling identified stone size as the most discriminative environmental variable, followed by stone height, vegetation coverage, and humidity, with temperature and vegetation height contributing minimally. These spatial differences likely reflect adaptations related to thermoregulation and predation (Ahnesjö and Forsman 2006). Arboreal *D. slowinskii* may depend on elevated canopy structure to regulate body temperature and capture invertebrate prey, consistent with patterns reported for *Saltuarius cornutus* (Gourret 2016), whereas saxicolous‐terrestrial *D. yangi* likely used rock and shrub cover for thermal buffering and predator avoidance, while foraging on ground‐dwelling arthropods (Monasterio et al. 2009).

Habitat variation imposes strong selective pressures that drive morphological diversification, with structural differentiation emerging as a central component of microhabitat specialization (Muñoz et al. 2013; Hu et al. 2019). In this study, arboreal *D. slowinskii* exhibited markedly larger overall size, elongated limbs, and an extended tail relative to the more saxicolous‐terrestrial *D. yangi* . These patterns align with ecomorphological divergence reported across other *Diploderma* lineages occupying contrasting ecological niches, where arboreal and ground‐associated forms show pronounced differentiation in body architecture and locomotor design (Shi et al. 2022). In arboreal settings, long and robust limbs increase stride reach and enhance climbing efficiency, while powerful claws improve grip stability during vertical and oblique movements (Lammers and Zurcher 2011). An elongated, muscular tail stabilizes the body during rapid positional transitions and supports complex aerial adjustments, thereby improving prey capture and escape performance under three‐dimensional canopy conditions (Jusufi et al. 2008). Such trait combinations represent recurring adaptive solutions in arboreal lizard radiations across diverse biogeographic regions (Knapp et al. 2016; Arange Lozano 2020). In contrast, the smaller stature of *D. yangi* likely promotes agile maneuvering and effective concealment within low shrubs and rocky substrates, consistent with crevice‐oriented morphologies documented in other taxa that exploit structurally intricate ground‐level habitats (Vitt et al. 1997). Collectively, divergence in size, limb configuration, and tail structure between these *Diploderma* species reflects functional optimization for locomotor efficiency, substrate use, and balance control in distinct ecological contexts, consistent with broader evolutionary patterns observed in limb and body‐form evolution among southern African agamids and other structurally specialized lizard groups (Tan et al. 2020).

Habitat‐driven trophic divergence constitutes a pivotal axis of ecological adaptation, enabling species to optimize resource exploitation under spatially heterogeneous selection regimes (Staudacher et al. 2018). Differential foraging strategies and resource utilization patterns, shaped by local prey availability and species‐specific morphological constraints, enhance fitness and reproductive success by promoting dietary efficiency and minimizing interspecific competition (Higginson et al. 2015). Using eDNA metabarcoding, this study uncovered pronounced trophic differentiation between two ecologically proximate *Diploderma* species in the Nujiang River Basin. Notably, although both *D. yangi* and *D. slowinskii* consumed plant materials and insects of varying sizes, based on the proportion of insect taxa with relative abundance exceeding 5%, *D. yangi* exhibited a dietary preference toward smaller ground‐dwelling prey, such as Armadillidiidae (24.69%) and Lygaeidae (8.98%), whereas *D. slowinskii* displayed a dietary profile skewed toward larger taxa, including Tettigoniidae (15.01%) and Scutigeridae (10.87%). These dietary distinctions were accompanied by significant interspecific variation in trophic niche breadth. Trophic profiles aligned closely with morphological and habitat differences: *D. slowinskii* exhibited greater body mass, longer limbs, and presumably stronger jaw musculature, traits that confer enhanced capacity to capture and subdue larger prey items. Its canopy‐dwelling ecology likely increases encounter rates with larger, actively flying insects, such as Asilidae and Crabronidae, reinforcing selective pressure toward dietary specialization. In contrast, *D. yangi* occupied lower‐lying, structurally complex habitats characterized by shrub cover and rocky crevices, which support higher densities of small cryptic invertebrates such as Psychodidae and Tephritidae. These microhabitat constraints likely favor generalist foraging and enhanced maneuverability. A comparable pattern has been documented in *Anolis* lizards, where species occupying distinct vertical strata of the arboreal environment exhibit marked trophic divergence, shaped by morphological differentiation and variation in prey distribution across microhabitats (Stroud et al. 2023). This coupling of habitat structure, morphological specialization, and dietary composition exemplifies a broader ecological mechanism wherein environmental heterogeneity drives niche partitioning among closely related taxa (Kliemann et al. 2021). To refine understanding of this process in *Diploderma*, future investigations should incorporate quantitative assessments of invertebrate biomass and functional traits across habitats to quantify the causal relationship between resource availability and foraging strategies. Such integrative approaches would clarify the mechanistic link between ecological opportunity and adaptive divergence, providing a stronger empirical basis for modeling the coexistence dynamics of these species.

By systematically comparing the habitat characteristics, morphological traits, and dietary differences between *D. yangi* and *D. slowinskii*—two lizard species with distinct life forms—this study validates that fine‐scale environmental heterogeneity may serve as a key factor promoting adaptive divergence and driving parapatric coexistence between these two species. Given that parapatric coexistence patterns resulting from such coordinated differentiation are often regulated by multiple interacting factors, subsequent research should further elucidate the detailed characteristics of habitat utilization, morphological adaptation, and trophic niche differentiation across different life forms, thereby clarifying the specific mechanisms driving both the origin and maintenance of parapatrically coexisting species. The findings of this study provide a mechanistic framework for understanding how habitat heterogeneity drives adaptive divergence and facilitates species parapatric coexistence, offering reasonable theoretical inferences and support for the formation and maintenance mechanisms of parapatric coexistence. Furthermore, these findings contribute novel insights to biodiversity conservation research in the Hengduan Mountains. Future investigations should incorporate expanded cross‐seasonal sampling and supplement analyses with local arthropod reference samples to more accurately elucidate the underlying drivers of dietary preferences and to characterize the temporal dynamics of these preferences in relation to niche structure. Additionally, in‐depth surveys targeting zones of potential niche overlap should be conducted to test the relative importance of habitat selection versus interspecific competition in maintaining species boundaries.

## Author Contributions

**Yuhao Wen:** conceptualization (lead), data curation (lead), formal analysis (supporting), methodology (equal), validation (equal), visualization (supporting), writing – original draft (equal), writing – review and editing (equal). **Songwen Tan:** conceptualization (supporting), data curation (supporting), formal analysis (lead), methodology (equal), software (lead), validation (equal), writing – review and editing (equal). **Yihua Xiang:** conceptualization (supporting), data curation (supporting), formal analysis (lead), investigation (equal). **Wei Gao:** conceptualization (supporting), data curation (supporting), validation (equal), writing – review and editing (equal). **Peng Guo:** formal analysis (supporting), investigation (supporting), writing – review and editing (equal). **Bingjun Dong:** formal analysis (supporting), investigation (supporting), methodology (lead), software (supporting), visualization (supporting), writing – original draft (equal), writing – review and editing (supporting). **Yayong Wu:** conceptualization (lead), data curation (lead), formal analysis (supporting), funding acquisition (supporting), investigation (supporting), methodology (supporting), project administration (lead), software (supporting), visualization (supporting), writing – original draft (equal), writing – review and editing (supporting).

## Funding

This work was supported by the Ph.D. Fund Project of Yibin University, 2019QD13. National Natural Science Foundation of China (32130015, 31801980).

## Conflicts of Interest

The authors declare no conflicts of interest.

## Supporting information


**Table S1:** Habitat selection preferences of *Diploderma yangi* and *D. slowinskii*.
**Table S2:** Morphological Sexual Dimorphism within *Diploderma yangi* and *D. slowinskii*: An Intraspecific Analysis.
**Table S3:** Analysis of morphological differences between *Diploderma yangi* and *D. slowinskii*.
**Table S4:** Family‐level food composition, relative abundance, and mean body size of species for *Diploderma yangi* and *D. slowinskii*.

## Data Availability

All analytical methods employed in this study utilized open‐source software, and the related eDNA data have been publicly deposited in the National Center for Biotechnology Information under the accession number PRJNA1399861 (https://www.ncbi.nlm.nih.gov/sra/?term=PRJNA1399861). Morphological data and habitat data were both uploaded as attachments to the submission system.
