# Supplementary material for: Habitat Heterogeneity Drives Niche Partitioning and Morphological Divergence in Two Parapatric Diploderma Lizard Species
Source: Ecol Evol. 2026 Jul 6;16(7):e73982. doi: 10.1002/ece3.73982 (PMC13337213; doi:10.1002/ece3.73982)
Supplement: Supplementary file 1 — Table S1: Habitat selection preferences of Diploderma yangi and D. slowinskii. Table S2: Morphological Sexual Dimorphism within Diploderma yangi and D. slowinskii: An Intraspecific Analysis. Table S3: Analysis of morphological differences between Diploderma yangi and D. slowinskii. Table S4: Family‐level food composition, relative abundance, and mean body size of species for Diploderma yangi and D. slowinskii. [file ECE3-16-e73982-s001.docx]

**Supplementary table**

**Supplementary table 1** **Habitat selection preferences of *Diploderma yangi* and *D. slowinskii***

| **Species** | **Variable** | **Test** | **Statistic** | ***P*** |
| --- | --- | --- | --- | --- |
| *D. yangi* | Landscape habitat | Chi-square Test | 0.67 | 0.41 |
|  | Habitat bottom material | Chi-square Test | 5.49 | 0.02 |
|  | Discovery location | Chi-square Test | 14.90 | < 0.001 |
|  | Vegetation type | Chi-square Test | 2.34 | 0.31 |
|  | Temperature | Mann-Whitney U Test | 433.50 | 0.20 |
|  | Humidity | Mann-Whitney U Test | 372.50 | 0.83 |
|  | Perch height | Mann-Whitney U Test | 202.50 | 0.01 |
|  | Vegetation coverage | Mann-Whitney U Test | 393.00 | 0.55 |
|  | Vegetation height | Mann-Whitney U Test | 352.00 | 0.90 |
|  | Stone height | Mann-Whitney U Test | 361.50 | 0.99 |
|  | Stone size | Mann-Whitney U Test | 140.50 | < 0.001 |
| *D. slowinskii* | Landscape habitat | Chi-square Test | 0.46 | 0.50 |
|  | Habitat bottom material | Chi-square Test | 3.55 | 0.060 |
|  | Discovery location | Chi-square Test | 29.04 | < 0.001 |
|  | Vegetation type | Chi-square Test | 5.31 | 0.07 |
|  | Temperature | Mann-Whitney U Test | 481.00 | 0.07 |
|  | Humidity | Mann-Whitney U Test | 334.50 | 0.50 |
|  | Perch height | Mann-Whitney U Test | 109.00 | < 0.001 |
|  | Vegetation coverage | Mann-Whitney U Test | 416.00 | 0.49 |
|  | Vegetation height | Mann-Whitney U Test | 403.50 | 0.63 |
|  | Stone height | Mann-Whitney U Test | 528.50 | 0.01 |
|  | Stone size | Mann-Whitney U Test | 555.50 | 0.00 |

**Supplementary table 2 Morphological Sexual Dimorphism within *Diploderma yangi* and *D. slowinskii*: An Intraspecific Analysis**

| **Morphological trait** | **DY-F (*n* = 15)** | **DY-M (*n* = 15)** | ***F/Z*** | ***P*** | **DS-F (*n* = 15)** | **DS-M (*n* = 15)** | ***F/Z*** | ***P*** |
| --- | --- | --- | --- | --- | --- | --- | --- | --- |
|  | **Mean ± SD** | **Mean ± SD** |  |  | **Mean ± SD** | **Mean ± SD** |  |  |
| SVL | 62.89 ± 3.35 | 63.63 ± 4.67 | 0.25 | 0.62 | 83.89 ± 7.34 | 91.72 ± 9.38 | 6.49 | 0.02 |
| TL | 116.31 ± 12.3 | 124.68 ± 15.14 | 2.76 | 0.11 | 193.10 ± 9.47 | 209.51 ± 30.14 | -2.34 | 0.02 |
| AL | 29.70 ± 2.92 | 28.27 ± 3.50 | 1.49 | 0.23 | 39.67 **±** 5.03 | 42.94 ± 6.54 | 2.35 | 0.14 |
| HL | 18.94 ± 1.57 | 20.28 ± 1.40 | 6.08 | 0.02 | 25.36 **±** 1.90 | 28.61 ± 3.55 | -2.80 | 0.01 |
| HW | 13.27 ± 1.40 | 14.60 ± 0.90 | -2.59 | 0.01 | 17.30 **±** 1.46 | 19.79 ± 2.58 | -2.59 | 0.01 |
| HH | 8.47 ± 0.61 | 9.08 **±** 0.97 | 4.23 | 0.05 | 11.39 **±** 1.09 | 12.65 ± 1.09 | 10.18 | 0.00 |
| SNL | 14.33 ± 1.20 | 15.64 **±** 1.40 | 7.61 | 0.01 | 19.24 ± 2.00 | 21.28 ± 2.24 | 6.92 | 0.01 |
| FLL | 19.07 ± 1.58 | 19.99 **±** 1.45 | 2.73 | 0.11 | 24.49 **±** 1.88 | 26.87 ± 1.75 | 12.88 | 0.00 |
| HLL | 27.41 ± 1.50 | 28.72 **±** 1.96 | 4.24 | 0.05 | 37.66 **±** 2.61 | 40.52 ± 3.29 | 6.96 | 0.01 |
| BM | 7.44 ± 1.52 | 8.11 **±** 1.69 | 1.32 | 0.26 | 19.37 **±** 4.39 | 25.37 ± 7.38 | 7.33 | 0.01 |

DY-F: *D.* *yangi* females; DY-M: *D.* *yangi* males; DS-F: *D.* *slowinskii* females; DS-M: *D.* *slowinskii* males*.* Abbreviations can be found in the Methods.

**Supplementary table 3 Analysis of morphological differences between *Diploderma yangi* and *D. slowinskii*.**

| **Morphological trait** | **DY-F (*n* = 15)** | **DS-F (*n* = 15)** | ***F*** | ***P*** | **DY-M (*n* = 15)** | **DS-M (*n* = 15)** | ***F*** | ***P*** |
| --- | --- | --- | --- | --- | --- | --- | --- | --- |
|  | **Mean ± SD** | **Mean ± SD** |  |  | **Mean ± SD** | **Mean ± SD** |  |  |
| SVL | 62.89 ± 3.35 | 83.89 ± 7.34 | 121.10 | < 0.001 | 63.63 ± 4.67 | 91.72 ± 9.38 | 114.30 | < 0.001 |
| TL | 116.31 ± 12.30 | 193.10 ± 9.47 | 300.90 | < 0.001 | 124.68 ± 15.14 | 209.51 ± 30.14 | 95.12 | < 0.001 |
| AL | 29.70 ± 2.92 | 39.67 **±** 5.03 | 71.10 | < 0.001 | 28.27 ± 3.50 | 42.94 ± 6.54 | 68.02 | < 0.001 |
| HL | 18.94 ± 1.57 | 25.36 **±** 1.90 | 100.40 | < 0.001 | 20.28 ± 1.40 | 28.61 ± 3.55 | 81.44 | < 0.001 |
| HW | 13.27 ± 1.40 | 17.30 **±** 1.46 | 59.50 | < 0.001 | 14.60 ± 0.90 | 19.79 ± 2.58 | 59.17 | < 0.001 |
| HH | 8.47 ± 0.61 | 11.39 **±** 1.09 | 91.20 | < 0.001 | 9.08 **±** 0.97 | 12.65 ± 1.09 | 82.19 | < 0.001 |
| SNL | 14.33 ± 1.20 | 19.24 ± 2.00 | 46.28 | < 0.001 | 15.64 **±** 1.40 | 21.28 ± 2.24 | 52.61 | < 0.001 |
| FLL | 19.07 ± 1.58 | 24.49 **±** 1.88 | 73.67 | < 0.001 | 19.99 **±** 1.45 | 26.87 ± 1.75 | 132.20 | < 0.001 |
| HLL | 27.41 ± 1.50 | 37.66 **±** 2.61 | 195.10 | < 0.001 | 28.72 **±** 1.96 | 40.52 ± 3.29 | 143.40 | < 0.001 |
| BM | 7.44 ± 1.52 | 19.37 **±** 4.39 | 140.20 | < 0.001 | 8.11 **±** 1.69 | 25.37 ± 7.38 | 110.70 | < 0.001 |

DY-F: *D.* *yangi* females; DS-F: *D.* *slowinskii* females; DY-M: *D.* *yangi* males; DS-M: *D.* *slowinskii* males*.* Abbreviations can be found in the Methods.

**Supplementary table 4 Family-level food composition, relative abundance, and mean body size of species for *Diploderma yangi* and *D. slowinskii***

| **Family** | **Frequency percentage (%)** | | | **Relative abundance (%)** | | **mean body size (mm)** |
| --- | --- | --- | --- | --- | --- | --- |
|  | **DY (n = 25)** | **DS (n = 10)** | | **DY** | **DS** |  |
| Acanthosomatidae | 0.20 | | 0.00 | 0.14 | 0.00 | 8 - 15 |
| Acrididae | 0.32 | | 0.00 | 0.24 | 0.00 | 15 - 45 |
| Alydidae | 0.20 | | 0.80 | 0.12 | 0.68 | 8 - 15 |
| Aphrophoridae | 0.24 | | 0.00 | 1.01 | 0.00 | 3 - 10 |
| Apidae | 0.00 | | 0.50 | 0.00 | 0.73 | 2 - 25 |
| Armadillidiidae | 0.80 | | 0.00 | 24.69 | 0.00 | 10 - 18 |
| Asilidae | 0.28 | | 0.90 | 0.24 | 5.88 | 4 - 26 |
| Attelabidae | 0.04 | | 0.60 | 0.00 | 3.80 | 2 - 15 |
| Carabidae | 0.48 | | 0.60 | 4.48 | 0.98 | 2 - 30 |
| Cerambycidae | 0.64 | | 0.30 | 0.98 | 0.00 | 5 - 40 |
| Ceratopogonidae | 0.08 | | 0.80 | 0.00 | 0.35 | 1 - 3 |
| Chironomidae | 0.40 | | 0.50 | 1.46 | 0.46 | 9 - 12 |
| Chrysomelidae | 0.56 | | 0.80 | 0.00 | 1.00 | 2 - 15 |
| Coccinellidae | 0.76 | | 0.30 | 1.05 | 0.00 | 0.8 - 18 |
| Coreidae | 0.00 | | 0.90 | 0.00 | 3.88 | 10 - 20 |
| Cosmopterigidae | 0.00 | | 0.50 | 0.00 | 0.38 | 3 - 8 |
| Crabronidae | 0.00 | | 0.30 | 0.00 | 5.13 | 5 - 20 |
| Crambidae | 0.68 | | 0.90 | 0.19 | 1.18 | 10 - 25 |
| Curculionidae | 0.44 | | 0.20 | 0.98 | 0.69 | 1 - 40 |
| Cydnidae | 0.20 | | 0.00 | 0.35 | 0.00 | 2 - 20 |
| Drosophilidae | 0.04 | | 0.50 | 0.00 | 2.78 | 2 - 4 |
| Elateridae | 0.84 | | 0.90 | 2.30 | 6.59 | 2 - 80 |
| Erebidae | 0.80 | | 0.00 | 8.98 | 0.00 | 10 - 35 |
| Formicidae | 1.00 | | 1.00 | 7.27 | 12.12 | 0.8 - 30 |
| Gelechiidae | 0.92 | | 0.80 | 1.41 | 0.00 | 5 - 15 |
| Geometridae | 0.60 | | 0.40 | 0.28 | 0.00 | 15 - 30 |
| Gracillariidae | 0.00 | | 0.40 | 0.00 | 2.47 | 3 - 8 |
| Gryllotalpidae | 0.28 | | 0.00 | 2.59 | 0.00 | 30 - 50 |
| Issidae | 0.04 | | 0.80 | 0.00 | 4.34 | 3 - 15 |
| Lycaenidae | 0.36 | | 0.10 | 1.37 | 0.00 | 15 - 30 |
| Lygaeidae | 0.88 | | 0.00 | 8.98 | 0.00 | 5 - 12 |
| Miridae | 0.32 | | 0.50 | 0.00 | 0.76 | 2 - 10 |
| Noctuidae | 0.84 | | 0.20 | 4.21 | 0.00 | 20 - 50 |
| Nolidae | 0.28 | | 0.80 | 0.19 | 1.91 | 15 - 35 |
| Nymphalidae | 0.28 | | 0.40 | 2.54 | 0.00 | 30 - 80 |
| Pentatomidae | 0.72 | | 0.70 | 4.24 | 7.73 | 10 - 30 |
| Phlaeothripidae | 0.24 | | 0.00 | 1.16 | 0.00 | 0.5 - 2 |
| Phoridae | 0.00 | | 0.70 | 0.00 | 0.11 | 1 - 4 |
| Pieridae | 0.52 | | 0.00 | 1.90 | 0.00 | 20 - 50 |
| Pipunculidae | 0.08 | | 0.10 | 0.00 | 0.56 | 1.5 - 8 |
| Pseudophyllodromiidae | 0.12 | | 0.00 | 0.13 | 0.00 | 10 - 50 |
| Psychodidae | 1.00 | | 0.90 | 4.47 | 1.60 | 1 - 5 |
| Sarcophagidae | 0.08 | | 0.80 | 0.00 | 2.53 | 8 - 25 |
| Scarabaeidae | 0.56 | | 0.60 | 2.09 | 0.00 | 5 - 50 |
| Sciaridae | 0.04 | | 0.80 | 0.00 | 0.43 | 1.5 - 5 |
| Scutigeridae | 0.32 | | 0.90 | 0.00 | 10.87 | 30 - 50 |
| Sesiidae | 0.32 | | 0.00 | 0.39 | 0.00 | 15 - 30 |
| Sphingidae | 0.40 | | 0.00 | 0.46 | 0.00 | 25 - 60 |
| Staphylinidae | 0.56 | | 0.40 | 0.97 | 0.00 | 1.5 - 25 |
| Syrphidae | 0.52 | | 0.60 | 0.00 | 0.11 | 4 - 15 |
| Tachinidae | 0.12 | | 0.80 | 0.19 | 0.33 | 5 - 25 |
| Tenebrionidae | 0.36 | | 0.90 | 0.20 | 3.47 | 5 - 30 |
| Tephritidae | 0.36 | | 0.20 | 2.88 | 0.00 | 5 - 15 |
| Tettigoniidae | 0.12 | | 0.80 | 0.00 | 15.01 | 11 - 87 |
| Thripidae | 0.16 | | 0.30 | 0.32 | 0.14 | 0.5 - 1.5 |
| Tortricidae | 0.40 | | 0.80 | 2.97 | 0.33 | 7 - 15 |
| Vespidae | 0.32 | | 0.00 | 0.24 | 0.00 | 5 - 30 |

DY: *D.* *yangi*; DS: *D.* *slowinskii.*
